# Medicated sex in Britain: evidence from the third National Survey of Sexual Attitudes and Lifestyles

**DOI:** 10.1136/sextrans-2015-052094

**Published:** 2015-06-19

**Authors:** Kirstin R Mitchell, Philip Prah, Catherine H Mercer, Jessica Datta, Clare Tanton, Wendy Macdowall, Andrew J Copas, Soazig Clifton, Pam Sonnenberg, Nigel Field, Anne M Johnson, Kaye Wellings

**Affiliations:** 1 Department of Social and Environmental Health Research, London School of Hygiene and Tropical Medicine, London, UK; 2 Research Department of Infection and Population Health, University College London, London, UK

**Keywords:** SEXUAL BEHAVIOUR, ERECTILE DYSFUNCTION, SEXUAL DYSFUNCTION, EPIDEMIOLOGY (GENERAL)

## Abstract

**Objectives:**

To describe the prevalence of medication use to assist sexual performance in Britain and to identify associated factors.

**Methods:**

Cross-sectional probability sample, undertaken in 2010–2012, of 15 162 people aged 16–74 years, resident in Britain, of whom, 5617 men and 8095 women reported sexual experience (ever) and 4817 men were sexually-active (reported sex in the last year).

**Results:**

Ever use of medication to assist sexual performance (medicated sex) was more commonly reported by men than women (12.9% (95% CI 11.9% to 13.9%) vs 1.9% (95% CI 1.7% to 2.3%)) and associated with older age in men and younger age in women. It was associated with reporting smoking, and use of alcohol and recreational drugs, as well as unsafe sex (≥2 partners and no condom use in the last year) in both men and women. Among men, the proportion reporting medicated sex in the last year was higher among those reporting erectile difficulties (ED) than those not doing so (28.4% (95% CI 24.4% to 32.8%) vs 4.1% (95% CI 3.4% to 4.9%)). In all men, medicated sex was associated with more frequent sexual activity, meeting a partner on the internet, unsafe sex and recent sexually transmitted infections diagnosis; associations that persisted after adjusting for same-sex behaviour and ED. However, there were significant interactions with reporting ED, indicating that among men with ED, medicated sex is not associated with same-sex behaviour and ever use of recreational drugs.

**Conclusions:**

A substantial minority of people in Britain report medicated sex, and the association between medicated sex and risky sexual behaviour is not confined to high-risk groups.

## Introduction

In recent years, there has been a proliferation of medication for sexual performance. Foremost are phospodiesteraise type 5 (PDE-5) inhibitors, which have revolutionised treatment of erectile difficulties (ED); Viagra (sildenafil citrate), in particular, has become a metaphor for male sexual prowess.^1^ Less well-known options for men include testosterone for low desire and selective serotonin reuptake inhibitors for early ejaculation. Medication for women mostly focuses on low sexual desire, and is limited in comparison. Prescribing of testosterone for women is highly restricted, and several drugs have recently failed to secure regulatory approval because their marginal benefits are outweighed by significant risk.^2^ Recreational drugs such as crystal methamphetamine are also used to facilitate arousal and promote more adventurous sexual activity,^3^ ^4^ often in combination with PDE-5 inhibitors.^5–7^

Use of medication may be formal or informal. Formal use implies treatment for a diagnosed sexual function problem under the care of a health professional, while the latter implies self-accessed medication or recreational drugs or off-label use of prescribed drugs, taken primarily to enhance sexual experiences. The informal use of pharmacological aids to sexual performance raises three concerns.^1^ ^8^ First, in specific population groups—including men who have sex with men (MSM),^4^ ^9–11^ undergraduate students,^7^ men entering HIV care^12^ and male sexually transmitted infections (STI) clinic attendees^13^—sildenafil citrate use is associated with unsafe sex and risk-related behaviours. Second, there are worries about potential iatrogenic effects, including harmful drug interactions between PDE-5 inhibitors and protease inhibitors and nitrates,^10^ ^14^ and erosion of confidence in ability to achieve an erection, creating psychological dependence.^15^ Third, where medication is accessed informally, there is no professional consultation to rule out contraindications or to ensure immediate and long-term safety for the individual. There is also a missed opportunity to talk to patients about their perceived need for medication, to discuss the implications for their partner^16^ and to consider possible alternative treatment options. As medications become easier to access without prescription, these issues are increasingly important. Formal use is of concern where treatment failure exacerbates low self-esteem caused by sexual dysfunction^17^ and where adverse side effects or health risks outweigh the benefits of use. Qualitative research suggests the possibility of significant drawbacks for partners and relationships; for instance, in reducing spontaneity and in creating pressure to have sex once an expensive pill has been taken.^16^ A number of vocal critics of the medicated sex phenomenon^18–20^ have argued that the increasing medicalisation of sex engendered by lifestyle drugs contributes to dissatisfaction with other than ‘perfect’ sex.^21^

Despite these significant concerns, little is understood about the phenomenon of medicated sex at a population level. The existing data are limited and come almost exclusively from convenience studies of specific population groups, particularly MSM.^4^ ^9^ ^10^ ^22^ ^23^ Such studies cannot tell us about the scale of use in the population as a whole, or whether associations with risk behaviour and low sexual function are confined to specific groups or apply more generally.

Here, we use data from a national probability sample, the third National Survey of Sexual Attitudes and Lifestyles (Natsal-3) to describe the use of any type of medication to assist sexual performance in the British population. We hypothesised that because of the relative lack of efficacious medication for women, more men than women would report medication use. We also hypothesised that, because of the salience and efficacy of PDE-5 inhibitors relative to other medication, men with ED would be more likely to use medication than those without, and the two groups would differ in terms of their health and level of sexual desire. Finally, we sought to determine whether associations between medication use and high levels of sexual behaviour and risk, same-sex sex and use of recreational drugs established by studies of high-risk groups, would also be observed at a population level.

## Methods

### Participants and procedure

Natsal-3 is a stratified probability sample survey of 6293 men and 8869 women aged 16–74 years, resident in Britain. The overall response rate was 57.7%, and the cooperation rate was 65.8% (of all eligible addresses contacted). Interviews were carried out between September 2010 and August 2012. Participants were interviewed using computer-assisted personal interviewing with computer-assisted self-interview (CASI) for the more sensitive questions. Details of the methods have been published previously.^24^ Natsal-3 was approved by the National Research Ethics Service Committee South Central—Oxford A (Ref: 10/H0604/27). Participants provided oral informed consent for interviews.

### Outcome measures

We asked sexually experienced participants—defined as those who reported ever having at least one sexual partner—about their use of medication to assist their sexual performance in the self-completion component of the questionnaire. The question was worded: ‘have you ever taken any type of medicine or pills to assist your sexual performance, for example Viagra? Include medication that has not been prescribed by a doctor’. Those who responded ‘yes’ were asked to report when they had last done so. Use of medication to assist sexual performance ever, and in the last year, was the primary outcome.

### Statistical analysis

We undertook all analyses using the complex survey functions of STATA V.12(Stata, College Station, Texas, USA) to incorporate the weighting, clustering and stratification of data. We present data on ever use among sexually experienced women and men. Analysis of medication use in the last year was restricted to sexually active men (defined as those reporting at least one sexual partner in the last year); the same analysis was not possible for women due to small numbers.

We used binary logistic regression to calculate ORs, adjusted for age, to examine the relationship between reporting medication use in the last year and a wide range of variables. To explore sociodemographic, health and sexual function variables, we investigated the use of medication separately among men who did and did not report ED in the last year. Men were asked if they had experienced any of a range of sexual problems for at least 3 months in the last year and those who endorsed the item ‘had trouble getting or keeping an erection’ were categorised as reporting ED in the last year. To understand how medication use varies with sexual lifestyles and behaviour, we explored associations between medication use and key sexual behaviour variables for all men, adjusting first for age, second for age and same-sex sex and third for age, same-sex sex and reporting ED. Item non-response was low (typically <3%).

## Results

### Medicated sex ever among sexually experienced men and women

Table 1 shows the proportion of men and reporting medicated sex ever, and factors associated with ever use. Among those sexually experienced (6863 men and 7067 women), ever use of medication was more commonly reported by men (12.9%, 95% CI 11.9% to 13.9%) than by women (1.9%, 95% CI 1.7% to 2.3%). We found opposite patterns of use by age; an association with older age in men and younger age in women (OR for 55–74 age group compared with 16–34 age group: 2.31 (95% CI 1.87 to 2.84) for men and 0.38 (95% CI 0.23 to 0.63) for women, p<0.001). In both men and women, medicated sex was significantly associated with relationship status. In men, after adjustment for age, medicated sex was associated with being in a steady relationship (but not living together) and with being previously in a live-in relationship. Among women, after adjustment for age, we found that medication use was less likely among single women, compared with those living with a partner. In both men and women, ever use of medication was associated with a range of health and lifestyle factors. We found associations with cigarette smoking, drinking more than 6–8 units of alcohol weekly and ever taking recreational drugs. In both genders, ever use of medication was associated with low overall sexual function in the last year. There was an association with reporting higher number of lifetime partners and with unsafe sex (reporting two or more partners, but no condom use in the last year), but these were stronger for men than for women (OR for unsafe sex 3.73 (95% CI 2.87 to 4.85) for men and 1.71 for women (95% CI 1.12 to 2.63), p<0.001). Finally, in both genders, reporting ever using medication was associated with having had a same-sex sexual experience.

**Table 1:** Prevalence of, and factors associated with, reporting ever use of medication to assist sexual performance by gender

	Men				Women			
	% reporting medication use	95% CI	Age-adjusted OR (95% CI)	Denominator: unweighted, weighted	% reporting medication use	95% CI	Age-adjusted OR (95% CI)	Denominator: unweighted, weighted
Overall	12.9	(11.9 to 13.9)		5617, 6863	1.9	(1.7 to 2.3)		8095, 7067
Age group								
16–34	9.2	(8.0 to 10.5)	1.00	2806, 2290	3.1	(2.6 to 3.8)	1.00	4092, 2269
35–54	11.8	(10.1 to 13.7)	1.32 (1.05 to 1.65)	1528, 2716	1.5	(1.1 to 2.1)	0.48 (0.32 to 0.71)	2235, 2774
55–74	18.9	(16.7 to 21.4)	2.31 (1.87 to 2.84)	1283, 1856	1.2	(0.8 to 1.9)	0.38 (0.23 to 0.63)	1768, 2023
Current relationship								
Living with a partner	12.5	(11.2 to 13.9)	1.00	2940, 4658	1.9	(1.6 to 2.4)	1.00	4356, 4673
In a steady relationship (but not living together)	13.9	(11.7 to 16.4)	1.66 (1.29 to 2.13)	955, 766	2.3	(1.5 to 3.4)	0.72 (0.44 to 1.18)	1373, 796
Previously in a live-in partnership	17.3	(14.5 to 20.5)	1.41 (1.10 to 1.81)	795, 708	2.1	(1.5 to 2.9)	1.25 (0.83 to 1.90)	1620, 1153
Not in a steady relationship	9.9	(7.6 to 12.8)	1.29 (0.91 to 1.81)	920, 725	1.1	(0.6 to 2.0)	0.31 (0.16 to 0.60)	738, 439
Smoke cigarettes nowadays								
No	11.9	(10.7 to 13.1)	1.00	3904, 5009	1.6	(1.3 to 1.9)	1.00	5803, 5355
Yes	15.5	(13.6 to 17.6)	1.60 (1.32 to 1.93)	1713, 1854	3.1	(2.5 to 4.0)	1.80 (1.30 to 2.50)	2292, 1711
Drink more than 6/8 units weekly								
No	12.3	(11.1 to 13.6)	1.00	4029, 5042	1.8	(1.5 to 2.2)	1.00	6273, 5543
Yes	15.9	(13.8 to 18.3)	1.47 (1.20 to 1.81)	1225, 1389	3.7	(2.6 to 5.1)	1.90 (1.27 to 2.85)	943, 772
Ever taken recreational drugs (not injected)								
No	10.6%	(9.5% to 11.9%)	1.00	3166, 4181	1.5	(1.2% to 1.9%)	1.00	5549, 5202
Yes	16.3%	(14.7% to 18.2%)	2.52 (2.07 to 3.07)	2445, 2677	3.1	(2.5% to 3.8%)	1.59 (1.14 to 2.22)	2542, 1862
Sexual function score in lowest quintile in the last year*								
No	10.5	(9.5 to 11.7)	1.00	3912, 4791	1.8	(1.5 to 2.2)	1.00	5465, 4628
Yes	22.9	(19.9 to 26.2)	2.33 (1.87 to 2.90)	937, 1199	3.5	(2.6 to 4.8)	2.20 (1.52 to 3.20)	1231, 1158
Number of sexual partners, ever								
1	6.0	(4.4 to 8.3)	1.00	765, 952	1.5	(1.0 to 2.4)	1.00	1606, 1613
2	6.6	(4.3 to 9.8)	1.14 (0.65 to 2.00)	478, 582	2.1	(1.2 to 3.5)	1.32 (0.64 to 2.71)	885, 813
3–4	8.2	(6.3 to 10.5)	1.44 (0.93 to 2.23)	861, 1058	1.4	(0.9 to 2.3)	0.88 (0.45 to 1.71)	1545, 1369
5–9	12.4	(10.5 to 14.5)	2.34 (1.58 to 3.45)	1391, 1718	1.2	(0.8 to 1.8)	0.68 (0.37 to 1.24)	2039, 1714
10+	19.2	(17.3 to 21.2)	4.06 (2.80 to 5.89)	2073, 2499	3.7	(2.9 to 4.6)	2.08 (1.27 to 3.42)	1958, 1502
2+ partners without using a condom, last year								
No	11.7	(10.7 to 12.8)	1.00	4894, 6200	1.8	(1.5 to 2.2)	1.00	7378, 615
Yes	25.4	(21.4 to 29.9)	3.73 (2.87 to 4.85)	611, 540	4.4	(3.0 to 6.3)	1.71 (1.12 to 2.63)	615, 7993
Ever had a same-sex experience								
No	11.9	(10.9 to 12.9)	1.00	5123, 6291	1.6	(1.3 to 1.9)	1.00	6937, 6213
Yes	23.8	(19.5 to 28.7)	2.33 (1.78 to 3.05)	494, 572	4.6	(3.4 to 6.1)	2.45 (1.69 to 3.56)	1158, 854

*Sexual function is measured by the Natsal-sexual function, which assesses problems with sexual response, sexual function in relationship context and self-appraisal of sex life.^25^

### Medicated sex in the last year among sexually active men

Table 2 shows associations between reporting medicated sex in the last year and demographic variables, health status, sexual function and help-seeking among men with and without ED in the last year.

**Table 2:** Prevalence of, and factors associated with, reported use of medication in the last year among sexually active men by reporting of erectile difficulties, no erectile difficulties and all*

	Men reporting erectile difficulties				Men not reporting erectile difficulties				All	
	% reporting medication use	95% CI	Age-adjusted OR (95% CI)	Denominator: unweighted, weighted	% reporting medication use	95% CI	Age-adjusted OR (95% CI)	Denominator: unweighted, weighted	% reporting medication use	**95% CI**
All										
	28.4	(24.4 to 32.8)		586, 766	4.1	(3.4 to 4.9)		4231, 5183	**7.2**	**(6.4 to 8.1)**
Age group										
16–34	13.2	(9.0 to 19.0)	1.00	208, 167	2.8	(2.2 to 3.6)	1.00	2438, 2000	**3.6**	**(2.9 to 4.4)**
35–54	31.6	(24.0 to 40.4)	3.04 (1.72 to 5.38)	152, 260	3.9	(2.8 to 5.3)	1.41 (0.93 to 2.14)	1191, 2215	**6.8**	**(5.4 to 8.4)**
55–74	33.5	(27.2 to 40.3)	3.31 (1.95 to 5.61)	226, 339	7.2	(5.4 to 9.6)	2.70 (1.81 to 4.03)	602, 967	**14.0**	**(11.7 to 16.6)**
Current relationship										
Living with a partner	28.8	(23.8 to 34.4)	1.00	330, 558	4.0	(3.1 to 5.0)	1.00	2369, 3693	**7.2**	**(6.2 to 8.4)**
In a steady relationship (but not living together)	27.5	(19.3 to 37.4)	1.52 (0.86 to 2.70)	115, 89	4.3	(3.1 to 6.0)	1.70 (1.06 to 2.74)	831, 669	**7.1**	**(5.5 to 9.1)**
Previously in a live-in partnership	32.5	(21.3 to 46.2)	1.55 (0.79 to 3.04)	69, 63	5.9	(3.8 to 9.0)	1.73 (1.03 to 2.92)	376, 324	**10.1**	**(7.4 to 13.7)**
Not in a steady relationship	21.0	(12.2 to 33.8)	1.51 (0.68 to 3.35)	70, 55	3.5	(2.1 to 5.7)	1.90 (0.95 to 3.79)	654, 495	**5.1**	**(3.5 to 7.3)**
Health										
Bad/very bad	37.6	(21.6 to 56.9)	1.00	32, 44	2.5	(0.7 to 8.5)	1.00	101, 125	**11.2**	**(6.6 to 18.5)**
Fair/good/very good	27.9	(23.8 to 32.5)	0.74 (0.32 to 1.69)	553, 720	4.1	(3.5 to 4.9)	2.22 (0.59 to 8.27)	4137, 5065	**7.1**	**(6.2 to 8.0)**
Health and/or medical condition affecting sexual activity, last year										
No	20.6	(16.0 to 26.2)	1.00	332, 408	3.6	(2.9 to 4.3)	1.00	3748, 4546	**4.9**	**(4.2 to 5.8)**
Yes	37.3	(30.9 to 44.2)	2.01 (1.30 to 3.12)	254, 358	7.7	(5.5 to 10.7)	1.96 (1.32 to 2.92)	490, 644	**18.1**	**(15.1 to 21.5)**
Lacked interest in having sex for 3+ months, last year										
No	30.4	(25.7 to 35.5)	1.00	437, 583	3.7	(3.0 to 4.4)	1.00	3673, 4477	**6.7**	**(5.9 to 7.7)**
Yes	22.2	(15.5 to 30.6)	0.76 (0.46 to 1.26)	149, 183	6.9	(4.7 to 10.1)	1.87 (1.20 to 2.93)	558, 706	**10.0**	**(7.6 to 13.1)**
Severe symptoms of erectile difficulties, last year										
No	25.0	(20.8 to 29.7)	1.00	504, 658					**6.4**	**(5.7 to 7.3)**
Yes	48.4	(36.7 to 60.3)	2.60 (1.49 to 4.54)	81, 107					**48.4**	**(36.7 to 60.3)**
Sought professional help regarding your sex life, last year										
No	14.4	(10.9 to 18.7)	1.00	430, 546	3.5	(2.9 to 4.3)	1.00	4053, 4964	**4.6**	**(3.9 to 5.4)**
Yes	63.3	(54.6 to 71.3)	9.42 (5.76 to 15.42)	156, 220	16.7	(11.3 to 23.9)	5.59 (3.46 to 9.02)	183, 222	**39.8**	**(34.1 to 45.8)**

*All participants have had at least one sexual partner in the past year.

Overall, 7.2% of men reported medicated sex in the last year. It was more frequently reported by men with ED (28.4%) compared with those without (4.1%). However, because only 13% of men reported ED in the last year (data not shown), at the population level, approximately 50% of medicated sex occurs among those without ED.

The prevalence of medicated sex in the last year increased with age in both groups of men. There was no association with partnership status in the ED group, but some evidence of an association in the group without ED; after adjustment for age, medication use in this latter group was less likely in those living with a partner. We found no association in either group with educational level or socioeconomic classification or with ethnicity or religious affiliation (data not shown).

In men with ED, as well as men without, we found no association between medication use and overall self-reported health, but a positive association with reporting a health condition that had affected sexual activity in the last year. Lack of interest in sex was associated with medication use, but only in men without ED (OR 1.87 (1.20 to 2.93)). For men with ED, symptom severity was strongly associated with medication use (OR 2.60 (1.49 to 4.54)). Seeking professional help for one's sex life was strongly associated with medication use in men with and without ED (OR 9.42 (5.76 to 15.42)) for men with ED and OR 5.59 (3.46 to 9.02)) for men without ED). Nonetheless, among men with ED, 14.4% of those who had not sought professional help in the last year had used medication (ie, medication use in absence of reported professional consultation).

Table 3 shows associations between reporting medication use in the last year and sexual behaviour and use of recreational drugs among all sexually active men.

**Table 3:** Prevalence of medicated sex in the last year, and sexual behaviours and recreational drug use associated with its use, among sexually active men

	All					
	% reporting medication use	95% CI	Age-adjusted OR (95% CI)	Adjusted OR* (95% CI)	Adjusted OR† (95% CI)	Denominator: unweighted, weighted
Same-sex identity and experience						
Sexual identity						
Heterosexual	7.0	(6.2 to 7.9)	1.00			4700, 5838
Any same-sex/other	13.9	(8.2 to 22.7)	2.57 (1.35 to 4.88)			161, 163
At least one same-sex sexual partner, past 5 years						
No	6.8	(6.0 to 7.7)	1.00			4685, 5825
Yes	17.9	(11.7 to 26.3)	3.40 (1.99 to 5.82)			180, 183
Sexual behaviour						
Had heterosexual sex before 16						
No	7.1	(6.1 to 8.1)	1.00	1.00	1.00	3368, 4342
Yes	7.5	(6.1 to 9.2)	1.31 (1.00 to 1.73)	1.30 (0.99 to 1.72)	1.40 (1.05 to 1.88)	1453, 1603
Number of sexual partners in the last year						
1	6.2	(5.4 to 7.2)	1.00	1.00	1.00	3568, 4823
2	7.7	(5.3 to 10.9)	1.94 (1.26 to 2.98)	1.86 (1.21 to 2.87)	1.80 (1.13 to 2.85)	539, 512
3+	14.4	(11.4 to 18.0)	4.44 (3.12 to 6.32)	3.92 (2.73 to 5.63)	3.63 (2.47 to 5.34)	719, 628
Number of occasions of sex, past 4 weeks						
0	5.0	(3.6 to 6.9)	1.00	1.00	1.00	1030, 1184
1–3	8.0	(6.6 to 9.6)	1.81 (1.20 to 2.72)	1.77 (1.17 to 2.66)	2.01 (1.31 to 3.09)	1617, 2155
4–7	6.9	(5.4 to 8.8)	1.79 (1.13 to 2.83)	1.74 (1.09 to 2.77)	1.85 (1.13 to 3.04)	1031, 1355
8+	7.2	(5.5 to 9.3)	2.22 (1.38 to 3.56)	2.13 (1.33 to 3.43)	2.76 (1.66 to 4.59)	1004, 1104
Masturbation in the last month						
No	6.6	(5.3 to 8.2)	1.00	1.00	1.00	1308, 1842
Yes	7.4	(6.4 to 8.6)	1.74 (1.28 to 2.37)	1.65 (1.21 to 2.25)	1.45 (1.06 to 2.00)	3545, 4152
Used internet to find sexual partner in past 12 months						
No	6.5	(5.7 to 7.4)	1.00	1.00	1.00	4512, 5683
Yes	18.9	(14.2 to 24.6)	4.70 (3.21 to 6.87)	4.00 (2.74 to 5.84)	3.63 (2.36 to 5.58)	352, 323
Paid for sex in the last year						
No	6.9	(6.1 to 7.8)	1.00	1.00	1.00	4798, 5929
Yes	28.7	(18.1 to 42.2)	5.67 (2.90 to 11.08)	5.18 (2.55 to 10.50)	5.22 (2.29 to 11.88)	66, 78
2+ partners without using a condom, last year						
No	6.4	(5.6 to 7.3)	1.00	1.00	1.00	4162, 5372
Yes	15.2	(11.9 to 19.2)	4.62 (3.25 to 6.56)	4.30 (3.03 to 6.12)	4.06 (2.80 to 5.90)	609, 537
Diagnosed with an STI, last year						
No	6.9	(6.1 to 7.9)	1.00	1.00	1.00	4755, 5895
Yes	22.4	(13.0 to 35.8)	7.81 (3.92 to 15.57)	6.52 (3.15 to 13.47)	5.02 (2.31 to 10.93)	72, 63
Recreational drug taking						
Ever taken recreational drugs (non-injecting)						
No	6.6	(5.6 to 7.7)	1.00	1.00	1.00	2621, 3535
Yes	8.1	(6.8 to 9.5)	2.39 (1.80 to 3.18)	2.26 (1.71 to 3.01)	2.34 (1.72 to 3.18)	2239, 2469
Ever taken amyl nitrates						
No	6.7	(5.9 to 7.6)	1.00	1.00	1.00	4456, 5546
Yes	12.8	(9.3 to 17.3)	3.19 (2.15 to 4.74)	2.75 (1.83 to 4.11)	2.60 (1.63 to 4.17)	404, 458

All participants have had at least one sexual partner in the last year**.**

*Adjusted for age and same-sex partnership in the past 5 years.

†Adjusted for age, at least one same-sex sexual partner in the past 5 years and reporting erectile difficulties.

STI, sexually transmitted infection.

Medication use was associated with higher levels of sexual activity, specifically reporting three or more partners in the last year and reporting eight or more occasions of sex in the past 4 weeks. We found strong associations with using the internet to find a partner in the last year, and with paying for sex. Unsafe sex (two or more partners and no reported condom use in the last year) and STI diagnosis in the past 5 years were also associated with medication use. After controlling for same-sex behaviour and reporting of ED, the associations between medication use and these variables remained significant, although most associations were slightly attenuated. Same-sex behaviour and ever use of recreational drugs such as amyl nitrates were associated with medication use, although we found significant interactions with reporting ED, indicating no association with same-sex behaviour and ever use of recreational drugs in men reporting ED.

## Discussion

We found that ever use of medication to assist sexual performance was much more common among men than women; men were over six times more likely to report ever having medicated sex (12.9% vs 1.9%). Although women reporting medicated sex tended to be younger and men to be older, ever use of medication was associated with recent use of other drugs (alcohol, cigarettes, recreational drugs), recent unsafe sex, same-sex sex and low overall sexual function in both genders. Use of medication in the last year is higher among men reporting ED, 28.4%, compared with 4.1% among men without difficulties, but at a population level, the proportion of medication users who do and do not report ED is similar. We found that recent use of medication increased with age in both groups of men, but low interest in sex was only associated with medication use in men without ED. Among men, medicated sex in the last year was associated with higher levels of sexual activity and a range of risk-related behaviours, and these associations remained after controlling for same-sex sex and reporting ED in the last year. Same-sex experience, as well as ever use of recreational drugs such as amyl nitrates, was significantly associated with medication use, but not in men reporting ED.

The strength of this study is that it is population-based, and provides, to our knowledge, the first generalisable estimates of medication use to assist sexual performance among British men and women. It augments studies of high-risk groups by establishing the strength of associations with key demographic, health and sexual behaviour variables at a population level. A limitation is that our outcome measure did not differentiate between formal and informal use; nor did it establish the type of medication most recently used. The question used the word ‘medicine’, and specified Viagra as an example, thereby strongly guiding participants towards medical rather than recreational drugs and towards erectile-dysfunction medication in particular. It, therefore, seems unlikely that many participants included recreational drugs such as amyl nitrates in their answers, particularly since, in a separate question few participants reported ever using them (table 3). Our finding of significantly higher prevalence of medication use among men with ED compared with men without strongly suggests that erectile-dysfunction medication was commonly reported. However, the outcome measure is harder to interpret with respect to women because much less is known about the types of medication commonly used by them. Our data are preliminary, and provide impetus for further research to explore medication use in women. The cross-sectional nature of the data means that it is not possible to ascertain the extent to which medication use facilitates risky behaviour or simply coincides with it.^26^ Finally, questions about sexual behaviour are sensitive, and use of medication might be prone to under-reporting. We sought to minimise this bias by the use of CASI.^24^

The gender difference in recent use of medication to assist sexual performance likely reflects the greater efficacy, wider availability and heavier marketing of medication for men relative to women. There is also perhaps a greater societal acceptance of pharmacological enhancement by men, concomitant with a greater pressure on men to demonstrate potency. The data suggest that the association with unsafe sex and STI diagnosis found for informal and recreational use of medication in selected high-risk populations^7^ ^10^ ^23^ is also evident at a population level, and for women as well as men. We found that associations with unsafe sex and high levels of sexual activity in men remained after controlling for same-sex behaviour, suggesting that the association between medicated sex and sexual risk is not restricted to those practicing same-sex sex. These findings suggest that medication use should be addressed in STI risk-reduction interventions; those targeting high-risk groups and those operating at a population level. In this study, over one in five men diagnosed with an STI in the last year also reported using medication, which suggests that STI diagnosis and treatment consultations would be an opportune moment for clinicians to discuss medication use with their patients.

In men without ED, the association between medication use and high levels of sexual activity on one hand, and low interest in sex on the other, seems paradoxical. Previous work sheds light on this apparent contradiction. Survey research shows an association between informal use of PDE-5 inhibitors and low erectile confidence,^15^ ^27^ and suggests that lack of sexual confidence is a common reason for use.^8^ Qualitative research suggests that informal medication use among highly sexually active homosexual men may be an attempt to mask a lack of sexual confidence and self-esteem^3^ or to mask low desire. It has also been suggested that informal users of PDE-5 inhibitors may develop unreasonable expectations of their erectile performance.^15^ Although the literature to date is largely focused on PDE-5 inhibitors, the risk that medication use leads to increasing dependence and decreasing confidence in one's own abilities to respond sexually is relevant to medical enhancement in general.

Our prevalence estimate of 14% among older men (aged 55–74) is identical to that estimated from a US probability sample survey of 3000 older adults (aged 57–85), which similarly asked participants about their use of ‘medication or supplements to improve sexual function’ in the last year.^28^ In addition, we found that 18% of men reporting same-sex experience in the past 5 years also reported performance-enhancing medication use in the last year, which is within the range of between 3% and 32% reported in a recent review of 11 studies of MSM.^23^

Primary care providers are accustomed to prescribing PDE-5 inhibitors for erectile dysfunction, but may not be aware of medication use in their other patients. Our data suggest that around 13% of their sexually-active male patients and 2% of their sexually-active female patients may have used medication to assist their sexual performance; about 4% of their male patients will be using medication for reasons other than ED, and 14% of their patients with ED who did not seek help in the last year will nonetheless be using medication. Where primary care and sexual health providers are aware of their patients’ use of medication, they can educate them about the risk of side effects, the implications for their partner and the potential for drug interactions. They may also be able to challenge beliefs that sex should be perfect and that medicine is required to reach perfection.^19^ ^21^ Such discussions could be framed by the wider context of a dramatic rise in lifestyle drugs to assist functioning in general.^1^ There may be a need for a public health campaign targeting men in the general population that encourages individuals to seek professional advice before taking medication.

This paper is among the first to address the evidence gap in our understanding of medicated sex at a population level. There is a need for further work to identify the range of medication in use, particularly among women. Qualitative research is also required to further understand the direction of causation between sexual risk taking and medication use. As medications become easier to access without prescription, it will be important to track how the association with risk varies with source of purchase. The scale of the phenomenon suggests that efforts to understand it should no longer be confined to studies of high-risk populations.

**Key messages**

- Ever use of medication to assist sexual performance is reasonably common in Britain, more so in men than women.
- It is associated with using other drugs, higher levels of sexual activity and unsafe sex in both women and men.
- In men, associations with high-risk behaviour persist after adjusting for same-sex behaviour, suggesting the association with high-risk is not restricted to those practicing same-sex sex.
- In men without erectile difficulties, medication use is associated with higher levels of sexual activity and also low interest in sex. This paradox is perhaps explained by low sexual confidence.
- Because medication is increasingly easy to access without prescription, there is a need for better professional and patient education on this phenomenon.
